# The Book World of Medicine and Science

**Published:** 1907-06-29

**Authors:** 


### The Book World of Medicine and Science.

Animal Micrology: Practical Exercises in Microscopical Methods. By M. F. Guyer, Ph.D. Chicago University Press. (London: T. Fisher Unwin, 1 Adelphi Terrace. 9s. net.)

There are already in existence so many practical textbooks on the microscope that it seems almost superfluous to increase their number. For all that Dr. Guyer has a right to claim that his handbook appeals to a class of student who finds the existing books either too bulky or too fragmentary. "Animal Micrology" hits, in most things, the happy mean. It tells a little of everything, and never tells so much of anything that the student becomes appalled by the magnitude of the task before him. Sometimes, indeed, it gives information in too condensed and concentrated a form. Thus, in a future edition, Dr. Guyer might with advantage amplify the short passages dealing with the use of the hæmocytometer, and might with equal advantage introduce the student to other methods in addition to the Thoma-Zeiss variety. In the chapter on stains no mention is made of Leishman's method, nor of Biebrich and Bremer's eosin stain. The book is not specially well adapted for the requirements of the clinical student: it contains much that he does not want and omits some points on which information would have been very useful to him. But for anyone who employs the microscope not merely on occasions when his clinical or post-mortem work requires it, but as a constant "hobby," the book is very suitable. It teems with practical hints such as can be offered only by one who has overcome difficulties himself. In the next edition the few printer's errors it contains may be corrected, and a couple of blank leaves might be inserted so as to give the student space for a few notes; at present the margin is not sufficiently broad to allow much note-taking.

